# Draft Genome Sequence of Pediococcus acidilactici Strain LPBC161, Isolated from Mature Coffee Cherries during Natural Fermentation

**DOI:** 10.1128/MRA.00332-19

**Published:** 2019-04-18

**Authors:** Elisângela S. M. Muynarsk, Gilberto V. de Melo Pereira, Dany Mesa, Vanete Thomaz-Soccol, Júlio C. Carvalho, Maria Giovana B. Pagnoncelli, Carlos Ricardo Soccol

**Affiliations:** a Department of Bioprocess Engineering and Biotechnology, Federal University of Paraná, Curitiba, Paraná, Brazil; b Department of Biochemistry, Federal University of Paraná, Curitiba, Paraná, Brazil; University of Rochester School of Medicine and Dentistry

## Abstract

Here, we report the draft genome sequence of Pediococcus acidilactici strain LPBC161, a lactic acid bacterium isolated from mature coffee cherries in Brazil. The genome sequence of *P. acidilactici* LPBC161 provides valuable information on the mechanisms of adaptation and metabolism of lactic acid bacteria (LAB) in the environment and stressor factors of coffee processing.

## ANNOUNCEMENT

Mature coffee cherries contain nutrients that favor the growth of lactic acid bacteria (LAB), which rapidly proliferate and initiate desirable lactic fermentation. These lactic acid-producing bacteria contribute to the demucilage process of coffee pulp, which is necessary for storage and transport of the coffee beans (1). The draft genome sequence of a LAB, isolated from coffee fruits in Brazil, was sequenced and analyzed. The isolate was obtained by plating depulped coffee fruits, collected during natural fermentation, onto MRS agar (2). Genomic DNA was extracted using the phenol-chloroform method (3) and was used in a PCR for amplification of the 16S rRNA gene according to the method described by Cruz et al. (4). The isolate was identified as Pediococcus acidilactici and named LPBC161.

The genomic DNA was quantified by Qubit (Invitrogen) fluorimetry, diluted, and used for the construction of genomic DNA sequencing libraries using a Nextera XT DNA sample preparation kit (Illumina), according to the manufacturer’s instructions. Final library quality control was performed using the 2100 bioanalyzer (Agilent Technologies). The libraries were diluted to 500 pM and pooled. This pool was quantified by quantitative PCR (qPCR) using a Kapa Biosystems preparation kit, and a 17.5 pM pooled library was sequenced with the Illumina MiSeq 500 v2 platform (paired-end, 250-bp reads). Overall, 5,795,728 reads were obtained, giving a 32-fold coverage for the strain sequenced. The Illumina reads were subjected to adapter trimming and quality filtering by Trimmomatic (5) with the following parameters: read value, <Q20; and read length, 150 bp. The quality of trimmed reads was checked by FastQC (www.bioinformatics.babraham.ac.uk/projects/fastqc/). The sequence data set was *de novo* assembled using the SPAdes program version 3.12 (6) with the careful mode enabled. Afterward, the genome sequences were submitted to the Rapid Annotations using Subsystems Technology (RAST) server annotation pipeline (7) to identify putative coding sequences (CDS) and provide an initial automatic annotation. When necessary, annotations were manually curated using Artemis (Sanger Institute, Cambridge, UK).

The draft genome of *P. acidilactici* LPBC161 was 1,960,506 bp, with a G+C content of 42.2% and *N*_50_ value of 18,140 bp. The genome annotation identified 2,019 coding sequences (CDS). Based on the Kyoto Encyclopedia of Genes and Genomes (KEGG) annotation, the core genome consists, in large part, of CDS for enzymes, transporters, and transcription factors. The LPBC161 strain can be classified as a facultative heterofermentative bacterium, possessing all enzymes required for glycolysis, phosphoketolase, and pentose-phosphate pathways. A genome comparison by BLASTn showed high identity with *P. acidilactici* SRCM100424 and *P. acidilactici* SRCM101189. Seventy-eight unique genes, including genes related with the phosphoenolpyruvate (PEP)-dependent phosphotransferase system and transcription regulation, were reported. *P. acidilactici* LPBC161 features a high number of specific genes encoding the PEP-phosphotransferase system for sorbose, mannose, and fructose, which are present in coffee pulp (8). In addition, coding sequences for enzymes involved in the use of various sugars are present, such as trehalose-6-phosphate hydrolase, alpha-l-rhamnosidase, 2-hydroxy-3-oxopropionate reductase, pyruvate kinase, glycerate kinase, beta-glucosidase, 6-phospho-beta-glucosidase, glucose-6-phosphate 1-dehydrogenase, 6-phosphofructokinase, galactosamine-6-phosphate isomerase AgaS, l-ribulose-5-phosphate 4-epimerase, and l-arabinose isomerase. *P. acidilactici* LPBC161 contains a range of genes encoding stress-related proteins, such as catalase, thiol peroxidase, glutathione reductases, NADH-oxidases, NADH peroxidises, F_o_F_1_-ATPase, and alkaline shock proteins. These data open new horizons for further research into the mechanisms of adaptation and metabolism of LAB and their influence on coffee processing and quality.

### Data availability.

The complete genome sequence for *P. acidilactici* LPBC161 has been deposited in DDBJ/ENA/GenBank under the accession number SAXR00000000. Raw sequencing data have been deposited in the SRA database under accession number SRX5328662. The genome sequence was also deposited at the National Center for Biotechnology Information (NCBI) under the BioSample accession number SAMN10690227 and assembly number ASM402229.
